# Standardized Testing, Use of Assessment Data, and Low Reading Performance of Immigrant and Non-immigrant Students in OECD Countries

**DOI:** 10.3389/fsoc.2020.544628

**Published:** 2020-11-26

**Authors:** Janna Teltemann, Reinhard Schunck

**Affiliations:** ^1^Department of Social Sciences, University of Hildesheim, Hildesheim, Germany; ^2^School of Human and Social Sciences, University of Wuppertal, Wuppertal, Germany

**Keywords:** immigration, education, standardization, PISA, educational inequality, principal-agent model, fixed effects, longitudinal analyses

## Abstract

This paper investigates the effects of standardized testing and publication of achievement data on low reading performance for immigrant and non-immigrant students in 30 OECD countries. The paper aims to test hypotheses derived from a principal-agent framework. According to this theoretical perspective, standardized assessments alone should not be associated with reading performance. Instead, the model proposes that the provision of the results to the principle (parents and education authorities) is associated with higher student performance, as this reduces the information asymmetry between principal (parents and educational authorities) and agent (teachers and schools). The results of our analyses of PISA 2009 and 2015 reading data from 422.172 students show that *first*, the use of standardized achievement tests alone was not associated with the risk of low performance. *Second*, making the results of standardized tests available to the public was associated with a decreased risk of low reading performance among all students, and, *third*, particularly among first generation immigrant students. These results were robust across various modeling approaches. In accordance with the predictions from the principal-agent framework, our findings suggest that the mere implementation of standardized assessments has no effects on low performance. Testing along with the public provision of the testing results, which decreases the information asymmetry between schools and teachers on the one hand and parents and education authorities on the other, was associated with a decreased risk of low performance, with the effect being stronger for immigrant students.

## Introduction

Integrating growing immigrant populations is a challenge for receiving countries. Since education is a key resource in contemporary societies it is also a key to societal integration of immigrants and, in particular, their descendants. International large scale assessments such as the OECD PISA study have drawn attention to countries' education systems and how they may contribute to educational inequalities and differences in integration processes. As pressure for quality and equity in education increased, policy making in education has been under close monitoring during the last years. A major focus of educational reform in many countries has been the implementation of educational standards and, in particular, their regular assessment through nationwide standardized testing (Scheerens, 2007; Meyer and Benavot, 2013). Standardized testing is supposed to aid the definition of clear educational goals and serves as a measure of accountability (i.e., the enforcement of responsibilities to attain these goals), which, in turn, are believed to affect incentives, restrictions, and opportunities of the actors involved in “producing” education. This rationale is drawn, in part, from principal-agent-models which are based in rational choice theories of individual action (Wößmann, 2005; Levačić, 2009). While principal-agent-models are often referred to in empirical research using large scale assessment data like PISA, their mechanisms are rarely put to a direct test. More often, these models are mentioned in order to explain a possible empirical association between standardized testing and educational outcomes.

In this paper, we add to the literature by, first, testing mechanisms drawn from a principal-agent model of education more directly. To do so, we investigate if the use of nationwide standardized testing affects student performance, and, more importantly, if reporting the results of such assessments to the public or educational authorities does. From the perspective of principal-agent models, we would expect that reporting of the results is particular important, since it reduces the information asymmetry between the agent (schools and teachers) and the principal (parents and educational authorities). Second, we take a closer look at immigrant students. The number of immigrants has increased substantially in most Western receiving countries during the last years. Third, because we focus on immigrant student, we do not examine average achievement as an outcome but the risk of low reading performance. This is defined as performance below the second proficiency level in reading in PISA. Reaching this level of reading proficiency is necessary to participate effectively in society and can thus be seen as a prerequisite for immigrant integration. Not reaching this level of proficiency is related to lower life chances: Follow-up studies based on PISA have shown that performance below this level is related to a lower chance of transition to post-secondary education and a higher risk of unemployment and income poverty (OECD, 2010; Shipley and Gluzynski, 2011). Fourth, we employ a longitudinal design at the country level by using data from the OECD Programme for International Student Assessment (PISA) 2009 and 2015 from 30 OECD countries. The longitudinal design allows us to control for (time constant) unobserved country characteristics, making the estimates less prone to bias.

The remainder of this paper is structured as follows: in the next section, we elaborate our theoretical arguments on the effects of standardized testing based on the principal-agent model. Thereafter, we summarize findings from previous studies on the impact of testing practices on educational outcomes. In section “Data and Methods” we describe our database and methods. After presenting the results in section “Results”, we discuss implications and limitations of our study in the final section.

## How Standardized Testing Can Affect Performance—Theory and Hypotheses

From a rational choice perspective, institutions of the education system affect incentives, restrictions, and opportunities of the actors involved (i.e., students, parents, teachers, principals). Following this rationale, education policies aiming for quality education should be most effective if they have implemented institutional regulations which incentivize high effort of the actors involved (e.g., teachers). Rational choice models of education further assume that actors, in our case teachers, may not necessarily be interested in high performance and may aim to avoid extensive effort. Parents and the state, however, expect schools and teachers to invest effort in teaching in order to realize quality education. This is a classic principal-agent constellation (Laffont and Martimort, 2002): A principal, the parents and/or the administrative authorities, commissions an agent, the school, to do something on their behalf, i.e., to provide education to the students (Ferris, 1992; Wößmann, 2005; Levačić, 2009).

The principal-agent framework draws attention to three possible problems (Jensen and Meckling, 1976): First, the agents' and principals' preferences may not align. Second, there is an asymmetry in information—oftentimes the principal cannot observe the agent's behavior directly. Third, the principal has to be able to evaluate the agent's behavior, i.e., he needs to assess how much effort the agent puts into realizing the principal's goals. Therefore, for principal-agent constellations to work in the principal's interest, at least two conditions have to be met. First, the principal's goals have to be clearly defined in order to be realized. This is one of the justifications for the specification of national standards in education. They are supposed to clarify the goals of education and function as a frame of reference and orientation for the actors involved (Klieme et al., 2003). Second, it is not sufficient to simply spell out the educational standards, they also need regular assessment. Hence, a frequently used indicator of the standardization of an education system is the use of regular (nation-wide) standardized tests.

A main argument in the literature is that standardized tests improve overall performance (Wößmann et al., 2009; Bol et al., 2014). The theoretical mechanisms governing this effect are however often rather implicit; mostly, it is assumed that the mere existence of such tests can either cause a form of “gentle pressure” on schools and teachers and their way of teaching or increase the signaling value of educational credentials (for a notable exception and an explicit theoretical model, see Bishop, 1995)^1^. It is argued, for instance, that if teachers do not know which tasks are assessed in tests—because the tests are conceptualized by a central authority—they will be less likely to skip parts of the curriculum and the content taught will be more comprehensive (Wößmann et al., 2007, p. 25f.).

However, from a theoretical point of view, this mechanism appears incomplete. The implementation of standardized testing itself is not sufficient to resolve the principal-agent problem, as it does not affect the information asymmetry between both parties. The principal needs to have information on the results of the standardized tests. The more information the principal has, e.g., achievement data of other schools or national averages, the better will the principal be able to evaluate the agent's behavior and sanction it, positively or negatively. Thus, only if the results of the standardized tests are available to the principal, will there be a relevant decrease in the asymmetric relation. From the logic of the principal-agent model, this form of accountability increases the agents' incentives to act according to the principals' preferences. Consequently, schools and teachers as agents are confronted with a higher pressure to improve their students' achievement. We therefore expect lower rates of low performing students in countries where assessment results are communicated to the public or administrative authorities (*Hypothesis 1*).

Furthermore, when it comes to the risk of low performance, different students have different risks. Immigrant students, for instance, are oftentimes in need of special individual (language) support. As their parents have less knowledge about the rules of the education system, teachers, and schools have to invest more time for consultation. The specific situation of immigrant students creates a higher demand for teachers and, from the perspective of the principal-agent model, a higher risk for opportunistic behavior (e.g., negligence of the specific needs of immigrant students). If, however, achievement data is available to the principals, this creates stronger incentives for schools to take care of every student, regardless of their background. The existence and publication of the results of standardized tests therefore should be advantageous for immigrants.

Further, we argue that it is rational for schools to concentrate efforts on those student groups who are in particular need for assistance (such as immigrant students) (Motiejunaite et al., 2014), as their performance may have a strong impact on a school's mean performance level. Findings from research on the effects of standardized assessments in the USA showed that for some tests and tasks, adaption of teaching strategies was more prevalent in schools with larger shares of ethnic minorities and low performing students (Mittleman and Jennings, 2018). Further, in some countries, standardized assessments are targeted toward minimum levels of education. As a consequence, teachers may particularly focus on students who are at risk of not reaching this level (Booher-Jennings, 2005), which often are immigrant or ethnic minority students. In the context of low educational performance, we thus expect immigrant students to profit more from standardized testing and a publication of assessment results than non-immigrant students (*Hypothesis 2*).

## Effects of Standardized Testing on Achievement—Previous Results

Since the publication of the first PISA round in 2000, a number of studies investigated how aspects of educational standardization are related to student achievement and inequality in student achievement (Schütz et al., 2007; Horn, 2009; Chmielewski and Reardon, 2016; Bodovski et al., 2017). These studies often focused on standardized testing, which is seen as one aspect of an education system's degree of standardization (Bol and van de Werfhorst, 2013). It has to be noted, however, that standardized testing should not be used alone to evaluate the degree of standardization of a country's education system. To assess if an education system can be described as standardized, other dimensions of (de)standardization, such as curriculum standardization, school autonomy (in selecting teachers, allocating resources, etc.), and the modes of teacher education, have to be considered as well. Since our focus lies on standardized testing—and not standardization in general—we concentrate the literature review on studies that either focus on this dimension or on immigrant students.

Several previous studies looked at the effect of central school exit exams, which are a special type of a standardized assessment, and mostly found that they are associated with higher average test scores (Bishop, 1997; Carnoy and Loeb, 2002; Wößmann, 2003; Fuchs and Wößmann, 2007). Bergbauer et al. (2018) compared the effects of standardized external comparisons and standardized monitoring to effects of more internal developed testing procedures, using data from six different PISA studies (2000–2015). Their results show that standardized external comparisons as well as standardized monitoring are associated with higher levels of competence among students. Drawing on data from TIMSS 1995, Jürges et al. (2005) analyzed the effect of central exit exams on achievement scores in lower secondary education in Germany. They found that students in federal states with central exit examinations outperform students in states without central school leaving assessments.

A small number of studies addresses the effects of testing on immigrant achievement and, to the best of our knowledge, there are no existing studies that focus on assessments and on the educational inclusion of immigrant students in terms of performance below a certain threshold. Schneeweis (2011) found significant (positive) effects of external student assessments on immigrants educational achievement only for OECD countries. Cobb-Clark et al. (2012) found insignificant effects of external examinations on test score gaps between immigrants and natives, only for one of eight assessed groups they estimated a significant negative effect. Teltemann (2015) found smaller achievement gaps in countries where accountability measures were implemented. Wößmann (2005) reported positive effects of central exams for low achieving students, suggesting that central exams bring an advantage for immigrant student and students from less-educated backgrounds.

## Data and Methods

We draw on data from the 2009 and 2015 OECD Programme for International Student Assessment (PISA, OECD, 2016). Both PISA rounds contain information on testing procedures and the publication of the testing results. Since its first survey in 2000, PISA is the most regular and wide-ranging competence assessment of secondary school students. In 2015, more than 540,000 students in 72 countries have been tested. PISA assesses curriculum-independent competences in reading, mathematics and science. In addition, PISA collects a broad range of background information by administering context questionnaires to students, parents, and principals. The sampling design is targeted at a representative sample of the 15-years old school population in a country, independent of the respective grade they are attending. PISA is conducted every 3 years and the PISA datasets are publicly available via download from the OECD's website^2^. Since we pooled the data from 2009 and 2015, we created a data structure with four levels: students, schools, country-years, and countries (see the section on Modeling below). All analyses were carried out using Stata 16.1. Code for reproducing the analysis have been archived on the Open Science Framework (https://osf.io/3ezxs/).

### Dependent Variable

With regard to immigrant integration, the definition of competences in PISA, which does not target national curricula but seeks to measure “viability” in globalized economies, proves useful. The PISA competence scores “measure how far students approaching the end of compulsory education have acquired some of the knowledge and skills essential for full participation in the knowledge society” (OECD, 2009b, p. 12). Thus, assessing differences between immigrant and non-immigrant students with PISA data can give insight not only into educational integration but also into future societal integration. Competences in PISA are measured on a continuous scale which is standardized to an OECD mean of 500 points. In addition, PISA distinguishes so-called proficiency levels, which correspond to actual abilities. For reading, proficiency level 2 is defined as a baseline level of competences, “at which students begin to demonstrate the reading skills that will enable them to participate effectively and productively in life” (OECD, 2016, p. 164). Performance below this baseline level thus indicates the risk of failed societal integration for immigrant students, as has been shown by PISA follow-up studies (OECD, 2010; Shipley and Gluzynski, 2011). PISA provides several (five up to 2012 and ten since 2015) plausible competence scores per student (see OECD, 2009a for details). We used the (first) five plausible values and created dummy variables that indicate performance below proficiency level 2 (a score below 408 points, see OECD, 2009a, p. 117ff.). Consequently, the final coefficients represent the average over five models (Macdonald, 2019).

### Main Independent Variable and Controls at the Student Level

In PISA, immigrant status is assigned according to the country of birth of a student and its parents. Students who indicated that they and their parents were born abroad are categorized as first generation students. Second-generation students were born in the country of test with both parents born abroad. Since PISA does not collect comparable or complete information on students' or parents' countries of origin—the way this is inquired differs between the participating countries—we cannot distinguish different immigrant groups. This is a major drawback, since the composition of immigrant groups may covary with the receiving countries' contextual conditions, including their educational institutions. To alleviate this problem partially, we control for language use at home with a dummy variable indicating whether students reported to mainly speak a foreign language and not the test language at home. Furthermore, because migration into OECD countries may be selective on socioeconomic status, we also control for several measures of parental socioeconomic background. This includes parental education (measured through the ISCED scale), family wealth possessions (measured through the “wealth” index in PISA), cultural possessions (measured through the “cultposs” index in PISA), and home educational resources (measured through the “hedres” index in PISA) (see OECD, 2017, p. 339 for details). Lastly, we control for student gender (1 = female).

### Main Independent Variables and Controls at the Country-Year Level

Following the approach described by the OECD (OECD, 2013, p. 28, 66, 166), we have aggregated school data within countries for 2009 and 2015 to describe the system level. This is possible since PISA draws a representative sample of schools and the schools' principals have been interviewed about organizational aspects of their school. For each year we constructed three variables according to this procedure: first, the proportion of students in a country attending schools that regularly administer mandatory standardized tests. Second, the proportion of students attending schools that post aggregated achievement data publicly and, third, provide aggregated achievement data to educational authorities^3^.

A country's institutional arrangements are not independent of other country characteristics that might also affect student achievement. Since we are employing a longitudinal approach at the country level and include country fixed effects (see Modeling below), all time-constant country differences are accounted for. However, effect estimates may still be biased by time-varying differences between countries that covary with standardized testing and student performance. We therefore control country characteristics that may simultaneously affect (immigrant) student performance and are related to the country's institutional arrangements. In order to control for a general effect of resources devoted to the educational system, we include annual educational expenditure as a percentage of a country's Gross National Income in our models. Likewise, we control for effects of economic development of a country by including the annual growth of a country's GDP (in percent). The overall number of immigrants in a country may be related to institutions, such as integration policies, which might have an impact on educational performance of immigrants. We therefore control for the international migrant stock as a percentage of the overall population. Additionally, immigrant performance may be impacted by their labor market outlooks. Hence, we control for the annual unemployment rate among foreign born persons in each country. Data for these annual country-year control variables comes from the World Bank and the OECD (Fontenay, 2018). An overview on the distribution of these characteristics among the countries in our sample can be found in the Appendix (Table A1) as well as their pairwise correlations (Table A3).

### Analyses Sample

We restricted our analyses to OECD countries in order to increase comparability across countries. We excluded countries for which (country-level) information was unavailable and those with <40 immigrant students (either first or second generation) in the sample—this applied to Japan, Korea, Poland, and Turkey. Students were excluded if they had missing values on any variable (listwise deletion). Our final sample consists of 422.172 students in 12.255 schools in 54 country-years in 30 countries. Table 1 gives an overview over unweighted sample statistics.

**Table 1:** Sample statistics (unweighted).

	**Mean**	**Sd**	**Min**	**Max**
**Student level variables**				
Below reading level 2 (pv1)	0.18		0.00	1.00
Below reading level 2 (pv2)	0.19		0.00	1.00
Below reading level 2 (pv3)	0.18		0.00	1.00
Below reading level 2 (pv4)	0.18		0.00	1.00
Below reading level 2 (pv5)	0.18		0.00	1.00
Native	0.89		0.00	1.00
First generation	0.05		0.00	1.00
Second generation	0.06		0.00	1.00
Gender [1 = female]	0.51		0.00	1.00
Language of test spoken at home	0.88		0.00	1.00
*Parental education*				
None	0.01		0.00	1.00
ISCED 1	0.03		0.00	1.00
ISCED 2	0.10		0.00	1.00
ISCED 3b,c	0.08		0.00	1.00
ISCED 3a,4	0.24		0.00	1.00
ISCED 5b	0.17		0.00	1.00
ISCED 5a,6	0.37		0.00	1.00
Index of family wealth possessions	−0.01	1.05	−7.44	4.44
Index of cultural possessions	−0.02	0.98	−1.92	2.63
Index of home educational resources	−0.05	1.00	−4.45	1.99
**Country level variables (source WB)**				
International migrant stock (% of population)	12.79	8.14	0.82	43.96
Adjusted savings: education expenditure (% of GNI)	5.03	0.93	3.10	8.34
GDP growth (annual, %)	−1.23	4.74	−14.43	25.16
Unemployment (%) among foreign born	11.63	6.18	4.30	32.00
*Proportion of students attending schools that (PISA aggr.)*				
Regularly use mandatory stand. tests	0.73	0.21	0.24	1.00
Post achievement data publicly	0.39	0.24	0.02	0.92
Provide adm. authority with achievement data	0.69	0.22	0.26	0.99
*PISA round*				
PISA 2009	0.58			
PISA 2015	0.42			
*N*	422,172			

*Source: PISA 2009, 2015, World Bank*.

### Modeling

As our dependent variable is binary and our data structure is clustered hierarchically, we estimated four level linear probability models (LPM). The individual students (level 1) are clustered in schools (level 2), which are clustered in country-years (triennial country observations) (level 3), which are again clustered in countries (level 4). The standard approach to this data structure is a four-level random effects model

(1) $$\begin{array}{l} y_{ijkl}=\beta_{0}+\beta_{1}x_{ijkl}+\beta_{2}c_{kl}+\beta_{3}x_{ijkl}\times c_{kl}+t+\;w_{l}+v_{kl}\\ \;+u_{jkl}+\varepsilon_{ijkl} \end{array}$$

where the dependent variable *y*_*ijkl*_ is the probability of an individual student *i* in school *j* in country-year *k* in country *l* to fall below PISA reading level 2. *w*_*l*_ represents the country-level error, *v*_*kl*_ the country-year error, *u*_*jkl*_ the school, and ε_*ijkl*_ the student-level error. *x*_*ijkl*_ exemplifies the individual-level variables (i.e., migration background, gender, language ability, and parental socio-economic status) and *t* represents joint period (wave) effects. The effects of interest are those associated with the country-year–specific variables (β_2_) and their interaction with immigration status (β_3_).

Although we focus on OECD countries, the country sample is still heterogenous with respect to immigration histories, institutional arrangements, educational policies, and economic conditions, all of which may be correlated with aspects of the education system and, in particular, testing and accountability. Thus, the problem of unobserved heterogeneity at the country level is pressing and the probability of misspecifying the model is high. The standard strategy to avoid misspecification is to control for the relevant confounders. However, the ability to include relevant confounders is restricted for two reasons. First, with 30 countries (and 54 country-years), the degrees of freedom are limited. Second, many important confounders, e.g., which describe a country's immigration history, are not readily measured and available. Therefore, we estimated (1) as a first difference (i.e., fixed effects) model (Wooldridge, 2010), including fixed effects for countries and years. The advantage of the fixed effects approach is that we do not have to make any assumptions about possible confounders at the country level. The model thus produces unbiased estimates even if there are unobserved confounders at the country level—that is, *E*(*w*_*l*_|*x*_*ijkl*_, *c*_*kl*_) ≠ 0. Therefore, the effects of the country-year level variables are estimated solely by relying on within-country (co)variation.

The coefficients in the LPM are estimators of the absolute difference in the probability of low reading achievement associated with a unit increase in the value of the corresponding predictor variable. We have chosen a linear probability model over a logistic model for the following reasons. First, the available non-linear four level models in the statistical program used for the analyses (Stata) do not accept weights. Weighting the data, however, is necessary in view of the complex and nationally diverging sampling procedures in PISA (OECD, 2009a; Lopez-Agudo et al., 2017). Second, non-linear models are notoriously hard to interpret, in particular when dealing with interactions. One needs to estimate average marginal effects in order to understand the joint effect of main- and interaction effect (Brambor et al., 2006; Berry et al., 2012). While other statistical software packages (e.g., MLwiN) are able to estimate weighted four level logit models, they are unable to provide average marginal effects. Third, an important argument against the LPM is that it may provide predicted probabilities >1 or <0 (Long, 1997). However, in many situations, the LPM is applicable (Hellevik, 2009) and, as the graphical illustration of the interaction effects below (**Figures 2**, **3**) show, predictions outside the range of 0 and 1 do not appear to be an issue here. Fourth, another argument against the LPM is that heteroscedasticity is almost inevitably present. For this reason and to account for the sampling (see below), we estimate robust standard errors. Nonetheless, to scrutinize the robustness of our analyses, we have additionally estimated standard logit models with cluster robust standard errors applying the same weights as for the LPMs (see Table A4 in the Appendix).

### Clustering, Standard Errors, and Weighting

PISA usually recommends to use balanced repeated replications (BRR) to estimate a coefficient's variance to take into account its complex sampling (OECD, 2009a; Lopez-Agudo et al., 2017). The particular variant used is known as Fay's method (Rust and Rao, 1996; Wolter, 2007). BRR breaks up the sample into subsamples (“replicates”) and the estimate of interest is first estimated for the full sample and then for each of the subsamples (Teltemann and Schunck, 2016). The estimator's variance is then estimated as the differences between the estimate from the full sample and each of the subsamples. We refrain from using BRR in this paper, because applying BRR may lead to a serious underestimation of the standard errors of country-level variables. Due to the resampling procedure, there will be no differences between the estimates for a country level variable in the full sample and the subsamples, because all students from one country have the same values for their country level variables.

Since the data is hierarchically structured with three clusters, it is necessary to account for the three-way clustering to estimate correct standard errors. Thus, we estimate cluster robust standard errors that account for the clustering at the country, the country-year, and the school level (Correia, 2017). Cluster-robust standard errors have shown to provide similar results for the lower level estimates when compared to BRR (Lopez-Agudo et al., 2017). To account for the complex sampling of PISA and the national differences in sampling, all analyses have been weighted by normalized student weights. In contrast to the final student weight, which is recommended for within-country analyses, applying these weights ensures that each country contributes equally to the analysis regardless of its actual size or student population.

## Results

**Figure 1:**
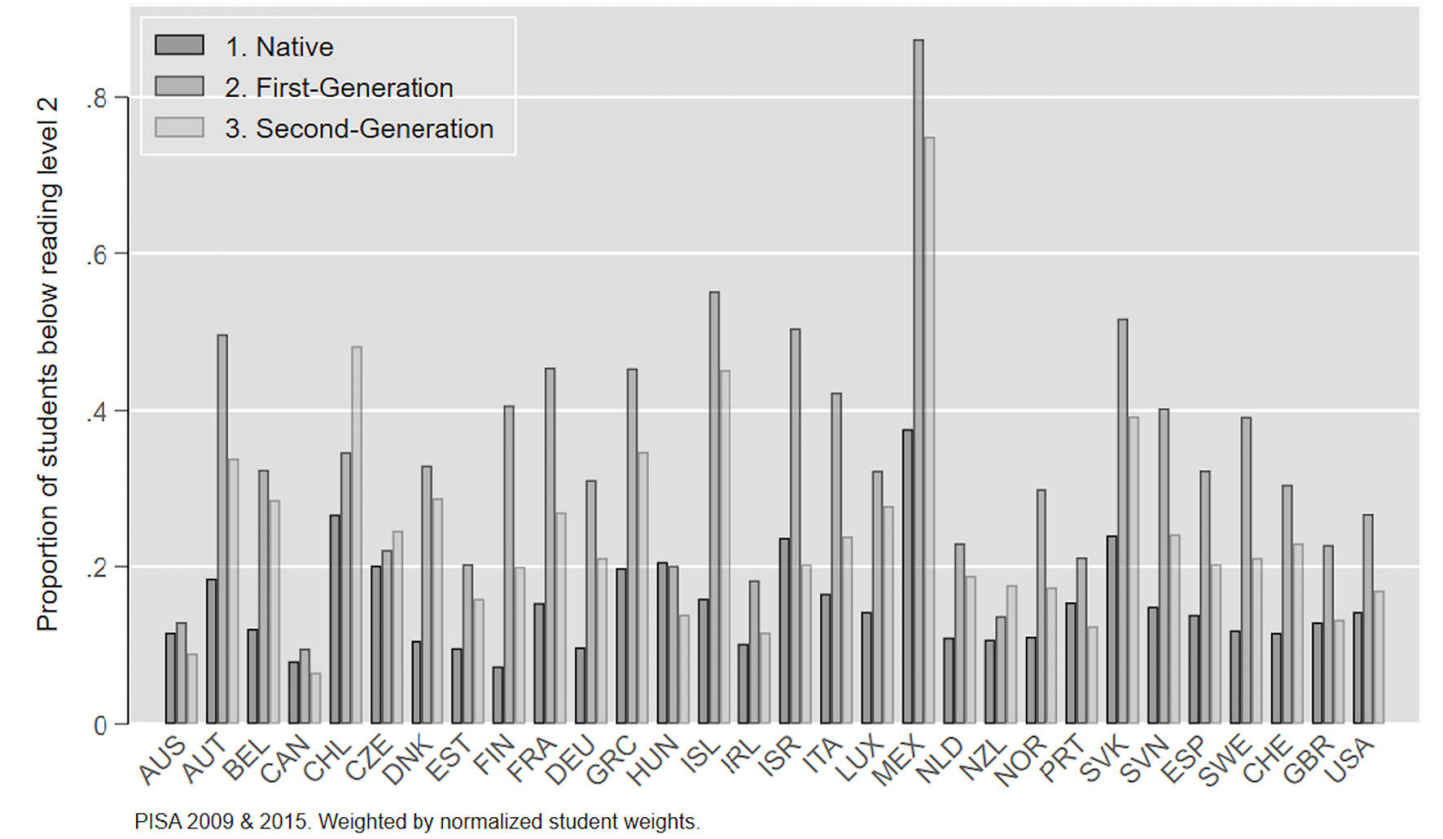
Proportion of students below baseline level in reading, PISA, 2009 and 2015.

Figure 1 shows the unadjusted risks for low performance among the different groups across the 30 countries in our sample averaged across 2009 and 2015. We see that first generation immigrants have a higher risk of performing below the baseline level of reading proficiency than non-immigrant students in most countries of our sample.

First generation immigrant students also have a higher risk of not reaching the baseline reading competence than second-generation immigrants in all countries except three (Chile, Czech Republic, New Zeeland). Second generation students generally still have higher risks of low performance compared to non-immigrants students with five exceptions (Australia, Canada, Israel, Hungary, Portugal), in which they show similar or lower risks than their fellow non-immigrant students.

Table 2 gives the results of our multivariate analyses. Model 1 includes only immigrant status and the country-level controls. It shows that first generation immigrants have a 16.1 percentage points higher probability of performing below the baseline level of proficiency than non-immigrants. Second generation immigrants have a 8.5 percentage points higher probability of low-performance than non-immigrants. After controlling for the individual-level characteristics (Model 2), the relatively higher risk for immigrants is reduced: Second generation immigrants only have about two percentage points higher risk of performing below the baseline level than non-immigrants, first generation immigrants still have about 9 percentage points higher risk. Model 3 includes the time-varying measure for the proportion of students attending schools that regularly employ standardized tests. While the estimated association is negative, statistical uncertainty is too high—the effect is not statistically significant. We also do not find statistically significant associations between the use of regular standardized tests and students' migration background (Model 4).

**Table 2:** Four level linear probability models predicting not reaching reading level 2.

	**1**	**2**	**3**	**4**	**5**	**6**	**7**	**8**
	**b/se**	**b/se**	**b/se**	**b/se**	**b/se**	**b/se**	**b/se**	**b/se**
**Student level**								
Native	ref.	ref.	ref.	ref.	ref.	ref.	ref.	ref.
First generation	0.161^***^	0.089^***^	0.089^***^	0.153^***^	0.089^***^	0.154^***^	0.089^***^	0.212^***^
	(0.021)	(0.017)	(0.017)	(0.037)	(0.017)	(0.019)	(0.017)	(0.039)
Second generation	0.085^***^	0.020	0.020	0.060	0.020	0.071^***^	0.020	0.107^**^
	(0.015)	(0.014)	(0.014)	(0.035)	(0.014)	(0.017)	(0.014)	(0.034)
Gender [1 = female]		−0.092^***^	−0.092^***^	−0.092^***^	−0.092^***^	−0.092^***^	−0.092^***^	−0.092^***^
		(0.005)	(0.005)	(0.005)	(0.005)	(0.005)	(0.005)	(0.005)
Language of test spoken at home		−0.094^***^	−0.094^***^	−0.093^***^	−0.093^***^	−0.095^***^	−0.094^***^	−0.094^***^
		(0.013)	(0.013)	(0.013)	(0.013)	(0.012)	(0.013)	(0.012)
*Parental education*								
None		0.056^**^	0.055^**^	0.055^**^	0.058^**^	0.058^**^	0.055^**^	0.055^**^
		(0.021)	(0.021)	(0.021)	(0.022)	(0.022)	(0.021)	(0.021)
ISCED 1		ref.	ref.	ref.	ref.	ref.	ref.	ref.
ISCED 2		−0.038	−0.039	−0.040	−0.038	−0.039	−0.040	−0.040
		(0.023)	(0.023)	(0.023)	(0.024)	(0.023)	(0.023)	(0.023)
ISCED 3b,c		−0.111^***^	−0.112^***^	−0.113^***^	−0.110^***^	−0.111^***^	−0.113^***^	−0.112^***^
		(0.025)	(0.025)	(0.025)	(0.026)	(0.025)	(0.025)	(0.025)
ISCED 3a,4		−0.152^***^	−0.153^***^	−0.154^***^	−0.151^***^	−0.152^***^	−0.153^***^	−0.153^***^
		(0.023)	(0.023)	(0.023)	(0.024)	(0.024)	(0.023)	(0.023)
ISCED 5b		−0.161^***^	−0.162^***^	−0.163^***^	−0.159^***^	−0.160^***^	−0.162^***^	−0.161^***^
		(0.023)	(0.023)	(0.022)	(0.023)	(0.023)	(0.023)	(0.023)
ISCED 5a,6		−0.176^***^	−0.177^***^	−0.177^***^	−0.175^***^	−0.175^***^	−0.177^***^	−0.176^***^
		(0.023)	(0.023)	(0.023)	(0.024)	(0.024)	(0.023)	(0.023)
Index of family wealth possessions		0.001	0.002	0.002	0.001	0.001	0.002	0.002
		(0.004)	(0.004)	(0.004)	(0.004)	(0.004)	(0.004)	(0.004)
Index of cultural possessions		−0.040^***^	−0.040^***^	−0.040^***^	−0.040^***^	−0.040^***^	−0.040^***^	−0.040^***^
		(0.004)	(0.004)	(0.004)	(0.004)	(0.004)	(0.004)	(0.004)
Index of home educational resources		−0.042^***^	−0.042^***^	−0.042^***^	−0.042^***^	−0.042^***^	−0.042^***^	−0.042^***^
		(0.004)	(0.004)	(0.004)	(0.004)	(0.004)	(0.004)	(0.004)
**Country-year level**								
GDP growth (annual, %)	−0.000	−0.001	−0.001	−0.001	−0.002	−0.002	−0.001	−0.001
	(0.002)	(0.001)	(0.001)	(0.001)	(0.001)	(0.001)	(0.002)	(0.002)
Education expenditure (% of GNI)	0.016	−0.008	−0.009	−0.009	−0.017	−0.016	−0.005	−0.003
	(0.015)	(0.012)	(0.012)	(0.012)	(0.012)	(0.012)	(0.019)	(0.019)
Migrant stock (% of population)	0.000	0.003	0.003	0.003	0.002	0.001	0.003	0.002
	(0.002)	(0.002)	(0.002)	(0.002)	(0.002)	(0.002)	(0.002)	(0.002)
Unemployment (%) among foreign born	0.001	0.000	0.000	0.000	0.001	0.001	0.000	0.000
	(0.001)	(0.001)	(0.001)	(0.001)	(0.001)	(0.001)	(0.001)	(0.001)
Proportion of student attending schools that			ref.	ref.	ref.	ref.	ref.	ref.
Regularly use mandatory stand. tests			−0.038	−0.033	−0.040	−0.041	−0.036	−0.036
			(0.036)	(0.035)	(0.033)	(0.034)	(0.035)	(0.035)
Prop. of schools X first gen.				−0.085				
				(0.056)				
Prop. of schools X second gen.				−0.053				
				(0.055)				
Post achievement data publicly					−0.158^*^	−0.144^*^		
					(0.067)	(0.069)		
Achievement data publicly X first gen.						−0.160^***^		
						(0.036)		
Achievement data publicly X second gen.						−0.124^***^		
						(0.029)		
Provide adm. authority with achievement data							0.029	0.051
							(0.101)	(0.099)
Achievement data adm. authority X first gen.								−0.179^**^
								(0.055)
Achievement data adm. authority X second gen.								−0.125^*^
								(0.049)
Country and year fixed effects	Yes	Yes	Yes	Yes	Yes	Yes	Yes	Yes
Constant	0.065	0.453^***^	0.488^***^	0.481^***^	0.600^***^	0.593^***^	0.443^*^	0.437^*^
	(0.067)	(0.070)	(0.081)	(0.081)	(0.087)	(0.088)	(0.174)	(0.172)
*N* countries	30	30	30	30	30	30	30	30
*N* country–years	54	54	54	54	54	54	54	54
*N* schools	12,255	12,255	12,255	12,255	12,255	12,255	12,255	12,255
*N* students	422,172	422,172	422,172	422,172	422,172	422,172	422,172	422,172

*Source: PISA 2009, 2015, World Bank. Standard errors in parentheses, adjusted for clustering in countries, country-years, and schools. Weighted by normalized student weights. Estimates averaged over five plausible values*.

* *p < 0.05*,

** *p < 0.01*,

*** *p < 0.001*.

In Models 5 and 7, accountability in terms of the provision of aggregated achievement data of schools to the general public (Model 5) or to administrative authorities (Model 7) is tested. Making achievement data available to the public is associated with a reduced probability of low reading performance among all students (b = −0.158, s.e. = 0.067, Model 5), while providing achievement data to administrative authorities is not associated with low reading performance (b = 0.029, s.e. = 0.101, Model 7). These findings thus only partly confirm the first hypothesis derived from the principal-agent framework.

**Figure 2:**
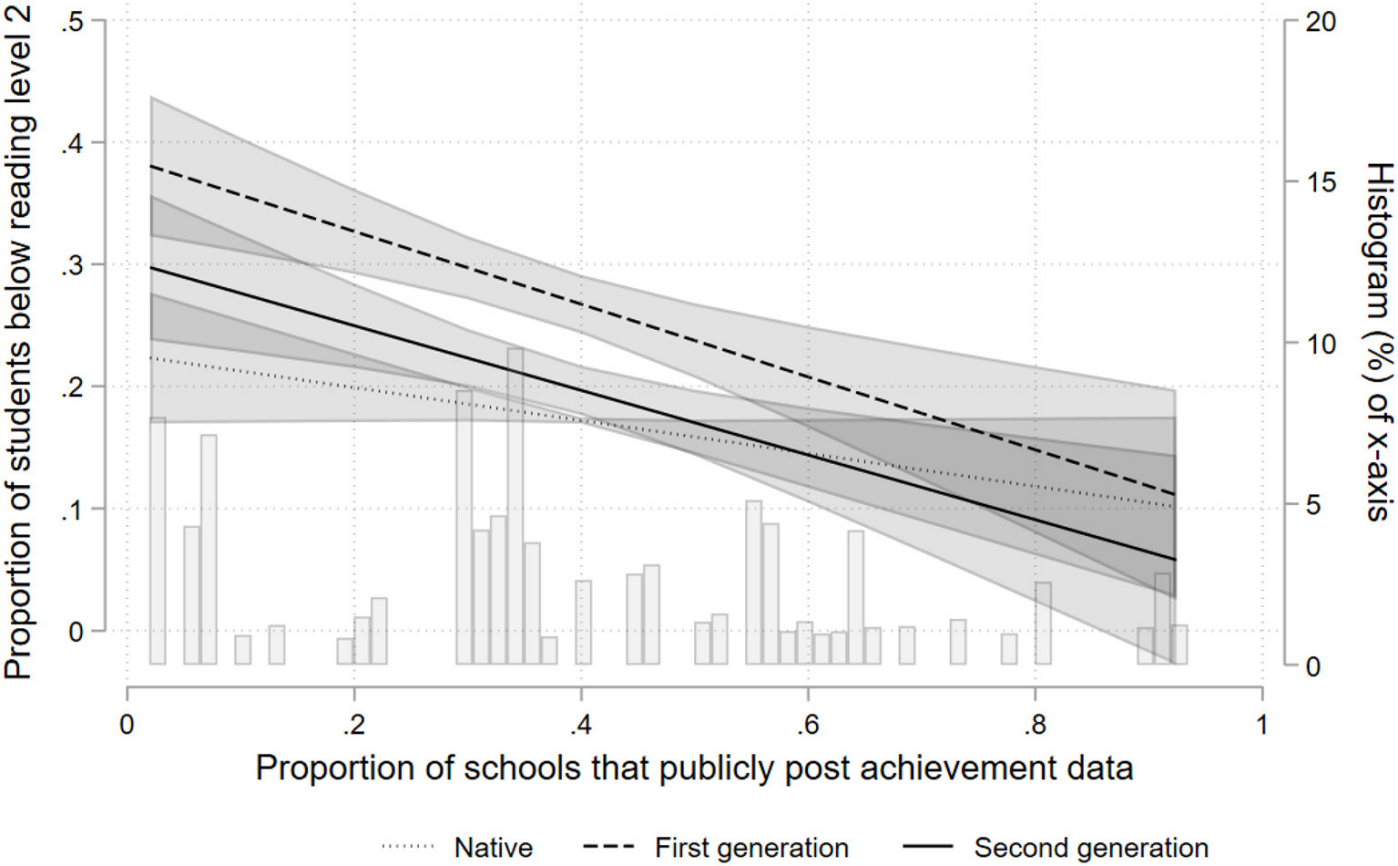
Probability of low performance according to accountability (data posted publicly).

**Figure 3:**
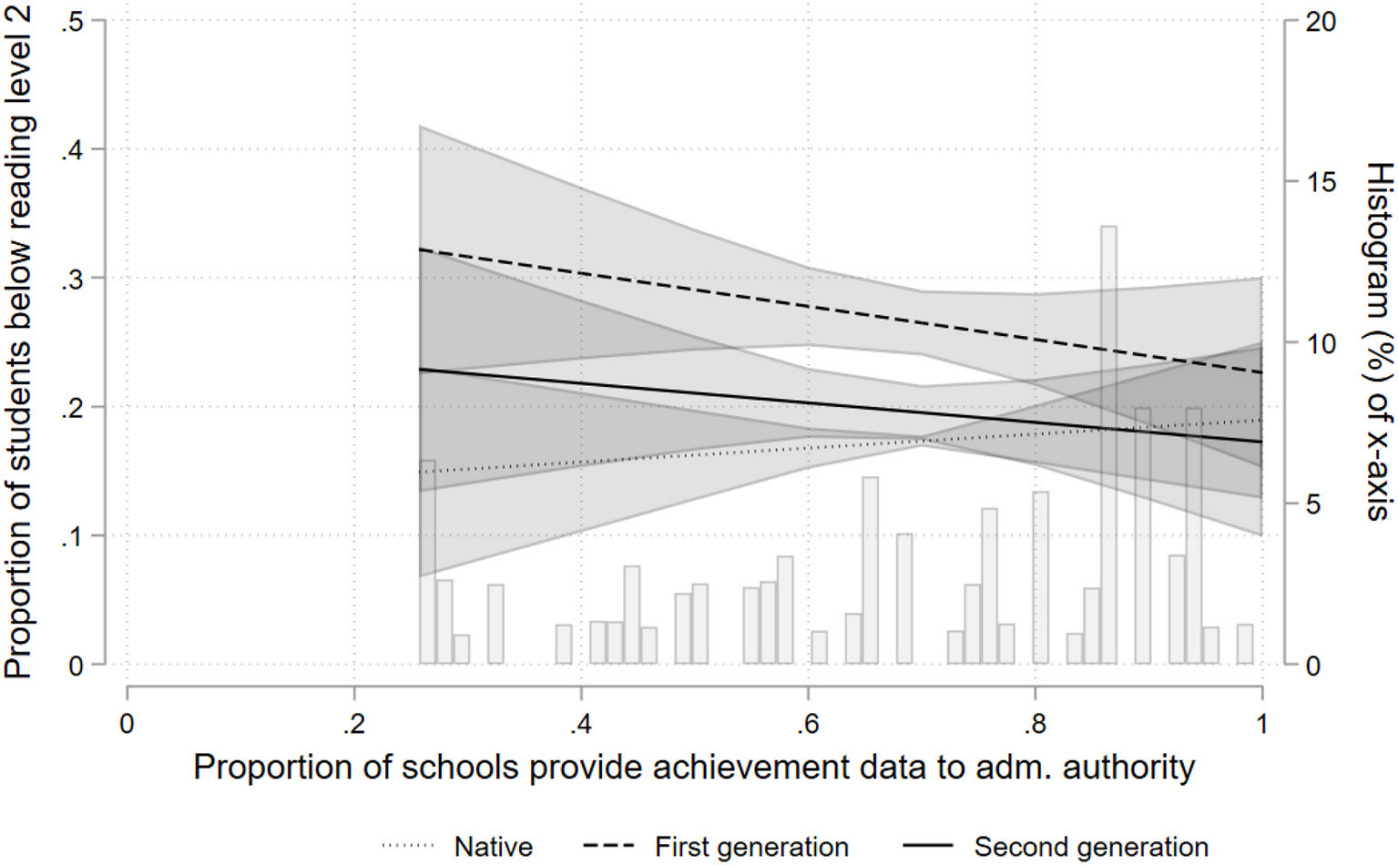
Probability of low performance according to accountability (data provided to administrative authorities).

Models 6 and 8 test the second hypothesis, which states that the communication of test results is expected to be associated with a reduced risk of low performance particularly among immigrant students. To facilitate interpretation, the Figures 2, 3 graphically display the interaction effects. The left y-axis shows the predicted probability of low performance based on the respective regression model. The scale of the left y-axis for each figure runs from 0.0 to 0.5; the figures thus cover a range of 50% points. The x-axis displays the proportion of students attending schools within a country which provide achievement data to the general public (or an administrative authority). The background of each figure additionally shows a histogram of the empirical distribution of the country-year level variable, that is the proportion of students that attend schools which provide information about achievement data to the respective recipient; this relates to the right y-axis. We limited the predicted values to an empirically reasonable range on the x-axis, i.e., for which we have observations in the data.

Figures 2, 3 show a similar pattern: The more prevalent accountability is in a country, the lower is the risk of low performance among immigrant students. Figure 2 shows a negative association between the public provision of aggregated achievement data and the risk of low reading performance for all students. The association is strongest for first generation immigrant students, reducing the risk of low performance by about 20 percentage points across the range of x. Figure 3 displays the estimated associations between the provision of aggregated achievement data to administrative authorities and the risk of low reading performance. There is a comparatively small effect for first generation immigrant students, about 9 percentage points across the range of x. While the association is also negative for second generation immigrant students, statistical uncertainty is high, as indicated by the large confidence intervals. The association for non-immigrant students appears slightly positive, but is far from statistical significance. Thus, the results are mostly compatible with our second hypothesis.

### Robustness Check

To see if the results of the analyses are sensitive to the modeling approach, we have estimated two sets of additional models. First, we have re-estimated all models as logit models with country and wave fixed effects and cluster robust standard errors, using the same weights as in the LPMs (see Table A4 in the Appendix). The results of the logit models support the conclusions drawn from the LPMs, with regard to the direction of the relevant coefficients and their statistical uncertainty. The logit models, too, estimate statistically significant, negative interaction effects, indicating that the provision of aggregated achievement data to the general public or to administrative authorities is associated with a reduced probability of low reading achievement among immigrant, in particular first generation, students. As in the LPMs, standardized testing alone is not statistically significantly associated with the risk of low reading performance—neither for immigrant nor for non-immigrant students. Second, we have re-estimated the models with the cross-level interaction as random effect models (with time fixed effect) and included random slopes for the interaction term. This may be necessary as leaving out a random slope for a cross-level interaction may cause the standard errors to be biased downwards (Heisig and Schaeffer, 2019). The results (see Table A5 in the Appendix) also support the conclusions drawn from the LPM. The provision of aggregated achievement data to the public or to administrative authorities is associated with lower probability of low reading achievement for immigrant students. However, statistical uncertainty for the latter association is too high, i.e., the interaction effects are not statistically significant.

## Discussion and Outlook

In this paper, we examined the effects of standardized testing and the publication of school achievement data on low reading performance for immigrant and non-immigrant students in 30 OECD countries using a longitudinal design at the country level by combining OECD PISA data from 2009 and 2015. We conceptualized low performance as the risk of performing below the so-called baseline level of reading proficiency in the PISA study (OECD, 2016, p. 164). With respect to immigrant students and their prospects for societal integration, performance above this baseline level is crucial, as it measures one's ability to fully participate in a society (OECD, 2009b, p. 2). We aimed at providing a more direct test for arguments drawn from the principal-agent models (William and Michael, 1976; Ferris, 1992; Laffont and Martimort, 2002), which are often mentioned in research on standardized testing and educational performance (Wößmann, 2005) but rarely directly tested.

Drawing on arguments from said principal-agent models, we hypothesized that standardized testing itself should not be sufficient to prevent low performance of students. We argued that an effect would only emerge if the principal, i.e., the administrative authorities or parents, had access to results of such testing. This would alleviate the information asymmetry between principal and agent, creating incentives for the agent (i.e., the school or the student) to prevent low performance. We furthermore expected immigrant students to profit more from this form of accountability than non-immigrant students, as they are often in need of special support.

The results of our analyses of PISA 2009 and 2015 reading data show that *first*, the use of standardized achievement tests alone was not associated with the risk of low performance. *Second*, making the results of standardized tests available to the public was associated with a decreased risk of low reading performance among all students, and *third*, particularly among first generation immigrant students. While the analyses also tended to confirm this relationship if the testing results were made available to an administrative authority, the estimated associations were smaller and not as robust. In a nutshell, the higher the share of schools that provide achievement data to the public, the lower is the risk for students, in particular for first generation immigrant students, to perform below reading level 2. These results were robust across the three modeling approaches we used: linear probability multilevel models with country and year effects and adjusted standard errors for multiple clustering (Wooldridge, 2010; Correia, 2017), linear probability models with year fixed effects and random slopes for the cross-level interactions (Heisig and Schaeffer, 2019) and cluster robust standard errors, as well as logit models with country and year fixed effects and cluster robust standard errors.

Overall, the results supported the hypotheses drawn from the principal-agent-model, as they showed that the mere existence of regular assessments is not sufficient to mitigate the information asymmetry between principal and agent if information from these assessments is not accessible. Assessments thus have to be combined with adequate measures of accountability in order to incentivize the actors to align their efforts with the principal's goals. The effects of assessments and accountability become especially apparent in the context of low performance and in particular for a specific group: immigrant students. We argued that assessments, which are often geared toward ensuring minimal levels of education, increase the incentives to support students at risk. As sufficient education is key for immigrant integration, education policies which lower the risk of low performance gain in importance.

### Limitations

Our study has several limitations that should be considered. First, the strength of international comparisons as we conducted it, is the variation in institutional characteristics. However, although all countries belong to the OECD, they are still heterogenous not the least with respect to their immigration history, which may be confounded with both educational institutions and (immigrant) student performance. We tried to approach this problem with a longitudinal approach at the country level, effectively controlling for all time-constant differences between countries, by focusing only on changes in the institutional arrangements within countries over time. Nonetheless, we only have two measurements over time. What is more, although we have tried to include the most relevant time-varying confounders at the country-year level, the estimated results are still prone to bias due to unobserved heterogeneity. Larger time-spans and additional meaningful controls at the country-year level would strengthen the analytical design. Second, it is unfortunate that PISA does not allow for a systematic and comparable differentiation of immigrant origin. We have attempted to alleviate this problem partially by controlling for different aspects of parental socio-economic status and language use at home. Still, we have to expect that the overall effect that we observed will vary across different countries of origin. However, the association is clearly present, even if the effect may be heterogenous across immigrant groups. Third, we have chosen a four-level linear probability model to analyze the data for the reasons outlined in the Data and Methods section, since the potentially better suited model (four level logit) could not be used. Nevertheless, comparisons of the LPMs' results with other modeling approaches (single level logit models and random intercept random slope models) showed very similar results. This increases our confidence that the results are not artifacts of the modeling approach. Fourth, the main proportion of variance in educational performance, including the risk of low performance, lies at the individual level. If we inspect empty random effect models, the intra-class correlations for the country and the country-year level are estimated to being only around 0.03. This has to be taken into consideration, when evaluating the results. The low intra-class correlation could be seen as an argument against investigating characteristics at the country(-year) level. Clearly, individual factors are responsible for the larger share of variation in educational performance. Nonetheless, we think that it is still relevant to analyze the role of institutional characteristics. From a policy perspective, institutional regulations are easier to adjust than students' characteristics. In a short term perspective, the latter has to be seen given. Profound knowledge about the effects—albeit small—of institutional characteristics of education system is crucial if one is interested in shaping institutions which facilitate sustainable development and system integration of contemporary societies. Fifth, although we tried to put the propositions of the principlal-agent framework to a direct test, we still face a black-box. With the data at hand, we do not know for certain if the mechanisms that create the association between (immigrant) student achievement and the public provision of assessment data correspond to those outlined in the principal agent framework. Further research could attempt to out even more specific hypotheses to the test. Our analyses fail to falsify predictions from the model, but should not be seen as a proof that the model is correct.

In summary, our results show that the mere implementation of standardized assessments has no effects on low reading performance, neither for immigrant nor for non-immigrant students. In line with the predications from a principal-agent framework, we do find a general association between provision of assessment data to the public and the risk for low reading performance. First generation immigrant students in particular have a reduced probability for low reading performance in countries that make assessment data available publicly.

## Data Availability Statement

Publicly available datasets were analyzed in this study. This data can be found at: https://www.oecd.org/pisa/data/2015database/; https://databank.worldbank.org/home.aspx.

## Author Contributions

JT and RS have jointly conceptualized and drafted the manuscript, approved it for publication, conceptualized the research question, and the theoretical approach. JT has conducted the literature review. RS conducted the empirical analyses. All authors contributed to the article and approved the submitted version.

## Conflict of Interest

The authors declare that the research was conducted in the absence of any commercial or financial relationships that could be construed as a potential conflict of interest.

## Footnotes

^1^One might argue that the signaling mechanism that is often referred to in the literature is very specific (Bishop, 1995). However, we do not know of any study using large scale assessment data, like PISA, which explicitly tests the mechanism, that is investigating if students really attach more value or importance to their education in the presence of standardized exit exams.

^2^https://www.oecd.org/pisa/data/

^3^As the sampling design of PISA targets the student population, not the schools in a country, computation of country level variables by aggregation has to be done with the (weighted) student level data. Since the sampling frames in PISA aim to provide representative information on all eligible students within a country, the resulting variables measure the proportion of students in a country attending schools with a respective feature (e.g., schools that make assessment data publicly available).
