# Supplementary material for: Standardized Testing, Use of Assessment Data, and Low Reading Performance of Immigrant and Non-immigrant Students in OECD Countries
Source: Front Sociol. 2020 Nov 26;5:544628. doi: 10.3389/fsoc.2020.544628 (PMC8022478; doi:10.3389/fsoc.2020.544628)
Supplement: Supplementary file 1 [file Table_1.docx]

**Table A1.** Country-samples with means for selected study variables (2009)

| Country | N | GDP growth (annual, %) | Education expenditure (% of GNI) | International migrant stock (% of population) | Unemployment (%) among foreign born | Prop. schools using stand. testing | Achievement data publicly | Achievement data adm. authority |
| --- | --- | --- | --- | --- | --- | --- | --- | --- |
| AUS | 13,176 | 1.940 | 4.800 | 26.54 | 6.700 | 0.700 | 0.470 | 0.810 |
| AUT | 5,799 | -3.760 | 5.550 | 15.20 | 9.500 | 0.320 | 0.060 | 0.490 |
| BEL | 7,377 | -2.020 | 6.250 | 9.630 | 16.20 | 0.270 | 0.020 | 0.450 |
| CAN | 21,574 | -2.930 | 4.580 | 20.55 | 10.10 | 0.880 | 0.550 | 0.890 |
| CHE | 10,518 | -2.220 | 4.580 | 26.50 | 6.900 | 0.670 | 0.030 | 0.330 |
| CHL | 5,351 | -1.560 | 4.230 | 2.170 | 8.200 | 0.860 | 0.350 | 0.770 |
| CZE | 5,701 | -4.800 | 3.970 | 3.790 | 9.600 | 0.890 | 0.310 | 0.560 |
| DEU | 3,928 | -5.700 | 4.650 | 14.43 | 13.10 | 0.400 | 0.110 | 0.290 |
| DNK | 5,192 | -4.910 | 7.960 | 9.180 | 9.900 | 0.970 | 0.450 | 0.560 |
| ESP | 24,626 | -3.760 | 4.440 | 13.48 | 27.40 | 0.290 | 0.080 | 0.650 |
| EST | 4,574 | -14.43 | 5.210 | 16.36 | 14.80 | 0.830 | 0.320 | 0.870 |
| FIN | 5,637 | -8.070 | 6.010 | 4.620 | 16.30 | 0.980 | 0.030 | 0.430 |
| FRA | -- | -- | -- | -- | -- | -- | -- | -- |
| GBR | 10,901 | -4.250 | 4.950 | 12.13 | 8.400 | 0.680 | 0.800 | 0.940 |
| GRC | 4,718 | -4.300 | 3.100 | 11.36 | 12 | 0.650 | 0.310 | 0.550 |
| HUN | 4,447 | -6.700 | 4.840 | 4.360 | 9.100 | 0.760 | 0.330 | 0.500 |
| IRL | 3,547 | -5.080 | 6.770 | 15.82 | 15.70 | 0.650 | 0.190 | 0.490 |
| ISL | 3,468 | -6.780 | 8.340 | 11.03 | 11.80 | 0.860 | 0.230 | 0.750 |
| ISR | -- | -- | -- | -- | -- | -- | -- | -- |
| ITA | 26,814 | -5.280 | 4.290 | 9.710 | 11 | 0.710 | 0.300 | 0.260 |
| LUX | 3,753 | -4.360 | 4.800 | 32.12 | 7.300 | 0.990 | 0.370 | 0.740 |
| MEX | 36,124 | -5.290 | 5.090 | 0.820 | 7 | 0.790 | 0.340 | 0.870 |
| NLD | 4,414 | -3.670 | 4.820 | 11.02 | 8.100 | 0.860 | 0.630 | 0.610 |
| NOR | 4,442 | -1.730 | 6.380 | 10.77 | 8.400 | 0.950 | 0.580 | 0.730 |
| NZL | 4,150 | -0.150 | 6.440 | 21.69 | 7 | 0.810 | 0.780 | 0.930 |
| PRT | 5,976 | -3.120 | 5.470 | 7.210 | 13.10 | 0.880 | 0.300 | 0.680 |
| SVK | 4,398 | -5.460 | 3.800 | 2.710 | 13.10 | 0.940 | 0.630 | 0.860 |
| SVN | 5,815 | -7.550 | 5.220 | 12.36 | 7.400 | 0.240 | 0.360 | 0.690 |
| SWE | 4,130 | -4.340 | 6.370 | 14.76 | 15.40 | 0.970 | 0.610 | 0.840 |
| USA | 4,952 | -2.540 | 4.710 | 14.26 | 9.700 | 0.970 | 0.890 | 0.950 |

Source: PISA 2009, 2015, World Bank.

**Table A2.** Country-samples with means for selected study variables (2015)

| Country | N | GDP growth (annual, %) | Education expenditure (% of GNI) | International migrant stock (% of population) | Unemployment (%) among foreign born | Prop. schools using stand. testing | Achievement data publicly | Achievement data adm. authority |
| --- | --- | --- | --- | --- | --- | --- | --- | --- |
| AUS | -- | -- | -- | -- | -- | -- | -- | -- |
| AUT | 6,723 | 1.010 | 5.210 | 17.47 | 10.70 | 0.640 | 0.060 | 0.640 |
| BEL | 8,943 | 2.030 | 6.120 | 12.28 | 17 | 0.420 | 0.030 | 0.590 |
| CAN | 18,590 | 0.660 | 4.930 | 21.80 | 7.500 | 0.840 | 0.570 | 0.930 |
| CHE | 5,579 | 1.330 | 4.470 | 29.39 | 7.900 | 0.620 | 0.080 | 0.440 |
| CHL | 6,734 | 2.300 | 4.570 | 2.620 | 5.800 | 0.980 | 0.530 | 0.860 |
| CZE | -- | -- | -- | -- | -- | -- | -- | -- |
| DEU | 5,248 | 1.740 | 4.380 | 14.88 | 7.700 | 0.420 | 0.140 | 0.380 |
| DNK | 6,773 | 2.340 | 6.900 | 10.10 | 12.20 | 0.890 | 0.450 | 0.750 |
| ESP | 6,340 | 3.840 | 4.040 | 12.69 | 29.80 | 0.460 | 0.210 | 0.810 |
| EST | 5,407 | 1.840 | 4.810 | 15.42 | 7.800 | 0.800 | 0.230 | 0.690 |
| FIN | 5,699 | 0.540 | 6.390 | 5.740 | 17.50 | 0.770 | 0.050 | 0.420 |
| FRA | 5,736 | 1.110 | 4.940 | 12.09 | 17.30 | 0.670 | 0.590 | 0.760 |
| GBR | 12,086 | 2.360 | 5.590 | 13.20 | 6.400 | 1.000 | 0.910 | 0.890 |
| GRC | 5,276 | -0.440 | 3.100 | 11.34 | 32 | 0.760 | 0.320 | 0.760 |
| HUN | 5,406 | 3.850 | 4.490 | 4.560 | 6.800 | 0.750 | 0.350 | 0.550 |
| IRL | 5,300 | 25.16 | 4.550 | 15.92 | 11.40 | 0.990 | 0.330 | 0.580 |
| ISL | 3,203 | 4.750 | 7.480 | 11.39 | 7 | 0.990 | 0.300 | 0.810 |
| ISR | 5,661 | 2.290 | 5.360 | 24.95 | 4.300 | 0.790 | 0.510 | 0.860 |
| ITA | -- | -- | -- | -- | -- | -- | -- | -- |
| ITA | 11,131 | 0.780 | 3.910 | 9.680 | 15.70 | 0.970 | 0.400 | 0.280 |
| LUX | 4,913 | 4.310 | 5.470 | 43.96 | 8.700 | 0.950 | 0.350 | 0.460 |
| MEX | 7,301 | 3.290 | 5.200 | 0.940 | 5.400 | 0.580 | 0.310 | 0.930 |
| NOR | 5,087 | 1.970 | 6.350 | 14.24 | 10.40 | 0.720 | 0.690 | 0.850 |
| NZL | -- | -- | -- | -- | -- | -- | -- | -- |
| PRT | 7,064 | 1.790 | 4.780 | 8.090 | 14.80 | 0.520 | 0.640 | 0.930 |
| SVK | 6,024 | 4.820 | 4.040 | 3.270 | 13.60 | 0.730 | 0.740 | 0.770 |
| SVN | 6,169 | 2.210 | 4.650 | 11.41 | 11.90 | 0.440 | 0.650 | 0.510 |
| SWE | 4,972 | 4.490 | 7.130 | 16.77 | 16.20 | 1.000 | 0.650 | 0.850 |
| USA | 5,305 | 2.880 | 4.400 | 14.49 | 5 | 0.920 | 0.920 | 0.990 |

Source: PISA 2009, 2015, World Bank.

**Table A3.** Correlations among country-level variables

|  | (1) | (2) | (3) | (4) | (5) | (6) | (7) |
| --- | --- | --- | --- | --- | --- | --- | --- |
| (1) GDP growth (annual, %) | 1.00 |  |  |  |  |  |  |
| (2) Adjusted savings: education expenditure (% of GNI) | 0.00 | 1.00 |  |  |  |  |  |
| (3) International migrant stock (% of population) | 0.25 | 0.05 | 1.00 |  |  |  |  |
| (4) Unemployment (%) among foreign born | -0.05 | -0.15 | -0.04 | 1.00 |  |  |  |
| (5) Proportion of students attending schools that (PISA aggr.) regularly use mandatory stand. tests | 0.14 | 0.15 | 0.01 | -0.46 | 1.00 |  |  |
| (6) Proportion of students attending schools that (PISA aggr.) post achievement data publicly | 0.18 | 0.01 | 0.00 | -0.38 | 0.53 | 1.00 |  |
| (7) Proportion of students attending schools that (PISA aggr.) provide adm. authority with achievement data | 0.10 | 0.15 | -0.06 | -0.19 | 0.26 | 0.60 | 1.00 |

Source: PISA 2009, 2015, World Bank

**Table A4.** Logit models with cluster robust standard errors predicting not reaching reading level 2

|  | 1 | 2 | 3 | 4 | 5 | 6 | 7 | 8 |
| --- | --- | --- | --- | --- | --- | --- | --- | --- |
|  | b/se | b/se | b/se | b/se | b/se | b/se | b/se | b/se |
| Student level: |  |  |  |  |  |  |  |  |
| Native | ref. | ref. | ref. | ref. | ref. | ref. | ref. | ref. |
|  |  |  |  |  |  |  |  |  |
| First generation | 1.045^***^ | 0.605^***^ | 0.607^***^ | 0.857^***^ | 0.611^***^ | 0.949^***^ | 0.607^***^ | 1.220^***^ |
|  | (0.083) | (0.082) | (0.082) | (0.181) | (0.081) | (0.078) | (0.082) | (0.165) |
| Second generation | 0.601^***^ | 0.158 | 0.159 | 0.332 | 0.157 | 0.480^***^ | 0.158 | 0.725^***^ |
|  | (0.085) | (0.093) | (0.093) | (0.238) | (0.093) | (0.118) | (0.093) | (0.215) |
| Gender [1=female] |  | -0.740^***^ | -0.740^***^ | -0.740^***^ | -0.740^***^ | -0.741^***^ | -0.740^***^ | -0.740^***^ |
|  |  | (0.044) | (0.044) | (0.044) | (0.044) | (0.045) | (0.044) | (0.045) |
| Language of test spoken at home |  | -0.626^***^ | -0.626^***^ | -0.623^***^ | -0.624^***^ | -0.636^***^ | -0.626^***^ | -0.631^***^ |
|  |  | (0.071) | (0.071) | (0.071) | (0.071) | (0.066) | (0.071) | (0.068) |
| Parental education |  |  |  |  |  |  |  |  |
| None |  | 0.200^*^ | 0.194^*^ | 0.194^*^ | 0.209^**^ | 0.212^**^ | 0.193^*^ | 0.194^*^ |
|  |  | (0.080) | (0.077) | (0.077) | (0.081) | (0.080) | (0.077) | (0.077) |
| ISCED 1 |  | ref. | ref. | ref. | ref. | ref. | ref. | ref. |
|  |  |  |  |  |  |  |  |  |
| ISCED 2 |  | -0.152 | -0.161 | -0.165^*^ | -0.152 | -0.158 | -0.162 | -0.163 |
|  |  | (0.085) | (0.085) | (0.083) | (0.089) | (0.086) | (0.084) | (0.084) |
| ISCED 3b,c |  | -0.533^***^ | -0.540^***^ | -0.544^***^ | -0.524^***^ | -0.529^***^ | -0.543^***^ | -0.542^***^ |
|  |  | (0.078) | (0.079) | (0.076) | (0.081) | (0.077) | (0.080) | (0.079) |
| ISCED 3a,4 |  | -0.809^***^ | -0.817^***^ | -0.822^***^ | -0.804^***^ | -0.814^***^ | -0.818^***^ | -0.820^***^ |
|  |  | (0.073) | (0.073) | (0.070) | (0.076) | (0.070) | (0.073) | (0.070) |
| ISCED 5b |  | -0.883^***^ | -0.890^***^ | -0.896^***^ | -0.873^***^ | -0.881^***^ | -0.891^***^ | -0.892^***^ |
|  |  | (0.070) | (0.070) | (0.066) | (0.070) | (0.065) | (0.070) | (0.067) |
| ISCED 5a,6 |  | -1.020^***^ | -1.028^***^ | -1.032^***^ | -1.014^***^ | -1.023^***^ | -1.028^***^ | -1.030^***^ |
|  |  | (0.069) | (0.070) | (0.067) | (0.073) | (0.067) | (0.070) | (0.067) |
| Index of family wealth possessions |  | 0.022 | 0.023 | 0.023 | 0.020 | 0.021 | 0.022 | 0.023 |
|  |  | (0.029) | (0.029) | (0.029) | (0.029) | (0.029) | (0.029) | (0.028) |
| Index of cultural possessions |  | -0.338^***^ | -0.338^***^ | -0.338^***^ | -0.338^***^ | -0.338^***^ | -0.338^***^ | -0.338^***^ |
|  |  | (0.027) | (0.027) | (0.027) | (0.027) | (0.027) | (0.027) | (0.027) |
| Index of home educational resources |  | -0.308^***^ | -0.307^***^ | -0.306^***^ | -0.309^***^ | -0.307^***^ | -0.307^***^ | -0.306^***^ |
|  |  | (0.024) | (0.024) | (0.024) | (0.024) | (0.024) | (0.024) | (0.024) |
| Country-year level: |  |  |  |  |  |  |  |  |
| GDP growth (annual, %) | -0.005 | -0.015 | -0.014 | -0.013 | -0.018 | -0.018 | -0.008 | -0.008 |
|  | (0.014) | (0.012) | (0.011) | (0.011) | (0.011) | (0.011) | (0.017) | (0.017) |
| Education expenditure (% of GNI) | 0.142 | -0.075 | -0.092 | -0.089 | -0.154 | -0.144 | -0.047 | -0.039 |
|  | (0.118) | (0.099) | (0.098) | (0.098) | (0.092) | (0.093) | (0.152) | (0.151) |
| Migrant stock (% of population) | -0.002 | 0.022 | 0.021 | 0.021 | 0.013 | 0.011 | 0.025 | 0.018 |
|  | (0.014) | (0.013) | (0.012) | (0.012) | (0.013) | (0.013) | (0.018) | (0.019) |
| Unemployment (%) among foreign born | 0.008 | 0.001 | 0.003 | 0.003 | 0.004 | 0.004 | 0.002 | 0.001 |
|  | (0.008) | (0.009) | (0.009) | (0.009) | (0.007) | (0.007) | (0.010) | (0.010) |
| Proportion of student attending schools that |  |  | ref. | ref. | ref. | ref. | ref. | ref. |
| regularly use mandatory stand. tests |  |  | -0.347 | -0.318 | -0.367 | -0.373 | -0.328 | -0.326 |
|  |  |  | (0.276) | (0.277) | (0.250) | (0.257) | (0.276) | (0.277) |
| prop. of schools X first gen. |  |  |  | -0.338 |  |  |  |  |
|  |  |  |  | (0.264) |  |  |  |  |
| prop. of schools X second gen. |  |  |  | -0.236 |  |  |  |  |
|  |  |  |  | (0.372) |  |  |  |  |
| post achievement data publicly |  |  |  |  | -1.304^*^ | -1.200^*^ |  |  |
|  |  |  |  |  | (0.525) | (0.529) |  |  |
| achievement data publicly X first gen. |  |  |  |  |  | -0.867^***^ |  |  |
|  |  |  |  |  |  | (0.187) |  |  |
| achievement data publicly X second gen. |  |  |  |  |  | -0.833^***^ |  |  |
|  |  |  |  |  |  | (0.234) |  |  |
| provide adm. authority with achievement data |  |  |  |  |  |  | 0.331 | 0.484 |
|  |  |  |  |  |  |  | (0.793) | (0.785) |
| achievement data adm. authority X first gen. |  |  |  |  |  |  |  | -0.907^***^ |
|  |  |  |  |  |  |  |  | (0.264) |
| achievement data adm. authority X second gen. |  |  |  |  |  |  |  | -0.843^*^ |
|  |  |  |  |  |  |  |  | (0.344) |
| Country and year fixed effects | ref. | ref. | ref. | ref. | ref. | ref. | ref. | ref. |
| Constant | -2.957^***^ | -0.814 | -0.464 | -0.504 | 0.658 | 0.645 | -1.071 | -0.992 |
|  | (0.425) | (0.475) | (0.520) | (0.522) | (0.675) | (0.674) | (1.621) | (1.602) |
| Wave: 2015 | -0.002 | 0.025 | 0.018 | 0.015 | 0.145 | 0.141 | -0.038 | -0.030 |
|  | (0.103) | (0.105) | (0.101) | (0.101) | (0.109) | (0.110) | (0.178) | (0.175) |
| N countries | 30 | 30 | 30 | 30 | 30 | 30 | 30 | 30 |
| N students | 422172 | 422172 | 422172 | 422172 | 422172 | 422172 | 422172 | 422172 |

Source: PISA 2009, 2015, World Bank. Standard errors in parentheses, adjusted for clustering in countries. Weighted by normalized student weights. * p<0.05, ** p<0.01, *** p<0.001.

**Table A5.** Four level linear probability models with random slopes predicting not reaching reading level 2

|  | 1 | 2 | 3 |
| --- | --- | --- | --- |
|  | b/se | b/se | b/se |
|  |  |  |  |
| Student level: |  |  |  |
| Native | ref. | ref. | ref. |
|  |  |  |  |
| First generation | 0.143^***^ | 0.164^***^ | 0.178^***^ |
|  | (0.036) | (0.021) | (0.044) |
| Second generation | 0.042 | 0.064^***^ | 0.074^*^ |
|  | (0.029) | (0.014) | (0.030) |
| Gender [1=female] | -0.085^***^ | -0.085^***^ | -0.085^***^ |
|  | (0.005) | (0.005) | (0.005) |
| Language of test spoken at home | -0.092^***^ | -0.092^***^ | -0.092^***^ |
|  | (0.011) | (0.011) | (0.011) |
| Parental education | ref. | ref. | ref. |
| None | 0.054^**^ | 0.054^**^ | 0.054^**^ |
|  | (0.018) | (0.018) | (0.018) |
| ISCED 1 | ref. | ref. | ref. |
| ISCED 2 | -0.035 | -0.035 | -0.035 |
|  | (0.022) | (0.022) | (0.022) |
| ISCED 3b,c | -0.102^***^ | -0.103^***^ | -0.102^***^ |
|  | (0.025) | (0.025) | (0.025) |
| ISCED 3a,4 | -0.132^***^ | -0.133^***^ | -0.132^***^ |
|  | (0.023) | (0.023) | (0.023) |
| ISCED 5b | -0.138^***^ | -0.139^***^ | -0.138^***^ |
|  | (0.023) | (0.023) | (0.023) |
| ISCED 5a,6 | -0.141^***^ | -0.142^***^ | -0.141^***^ |
|  | (0.023) | (0.023) | (0.023) |
| Index of family wealth possessions | 0.003 | 0.003 | 0.003 |
|  | (0.004) | (0.004) | (0.004) |
| Index of cultural possessions | -0.033^***^ | -0.033^***^ | -0.033^***^ |
|  | (0.003) | (0.003) | (0.003) |
| Index of home educational resources | -0.038^***^ | -0.038^***^ | -0.038^***^ |
|  | (0.003) | (0.003) | (0.003) |
| Country-year level: | ref. | ref. | ref. |
| GDP growth (annual, %) | -0.002^*^ | -0.002^*^ | -0.002^*^ |
|  | (0.001) | (0.001) | (0.001) |
| Education expenditure (% of GNI) | -0.010 | -0.010 | -0.010 |
|  | (0.006) | (0.005) | (0.005) |
| Migrant stock (% of population) | -0.003^**^ | -0.003^**^ | -0.003^**^ |
|  | (0.001) | (0.001) | (0.001) |
| Unemployment (%) among foreign born | -0.002 | -0.002 | -0.002 |
|  | (0.002) | (0.002) | (0.002) |
| Proportion of student attending schools that | ref. | ref. | ref. |
| regularly use mandatory stand. tests | -0.038 | -0.034 | -0.046 |
|  | (0.032) | (0.034) | (0.034) |
| prop. of schools X first gen. | -0.053 |  |  |
|  | (0.052) |  |  |
| prop. of schools X second gen. | -0.027 |  |  |
|  | (0.042) |  |  |
| post achievement data publicly |  | -0.010 |  |
|  |  | (0.037) |  |
| provide adm. authority with achievement data |  |  | 0.055 |
|  |  |  | (0.040) |
| achievement data publicly X first gen. |  | -0.154^***^ |  |
|  |  | (0.037) |  |
| achievement data publicly X second gen. |  | -0.106^***^ |  |
|  |  | (0.027) |  |
| achievement data adm. authority X first gen. |  |  | -0.111 |
|  |  |  | (0.069) |
| achievement data adm. authority X second gen. |  |  | -0.077 |
|  |  |  | (0.044) |
| Wave: 2015 | 0.015 | 0.016 | 0.011 |
|  | (0.011) | (0.012) | (0.012) |
| Constant | 0.550^***^ | 0.552^***^ | 0.521^***^ |
|  | (0.067) | (0.066) | (0.060) |
| Var(Countries) | 0.002^***^ | 0.002^***^ | 0.002^***^ |
|  | (0.000) | (0.000) | (0.000) |
| Var(Country-years) | 0.006^***^ | 0.004^***^ | 0.005^***^ |
|  | (0.001) | (0.001) | (0.001) |
| Var(First generation) | 0.001^***^ | 0.001^***^ | 0.001^***^ |
|  | (0.000) | (0.000) | (0.000) |
| Var(Second generation) | 0.001^***^ | 0.001^***^ | 0.001^***^ |
|  | (0.000) | (0.000) | (0.000) |
| Var(Schools) | 0.010^***^ | 0.010^***^ | 0.010^***^ |
|  | (0.002) | (0.002) | (0.002) |
| Var(Students) | 0.113^***^ | 0.113^***^ | 0.113^***^ |
|  | (0.003) | (0.003) | (0.003) |
| N countries | 30 | 30 | 30 |
| N country-years | 54 | 54 | 54 |
| N schools | 16854 | 16854 | 16854 |
| N students | 422172 | 422172 | 422172 |

Source: PISA 2009, 2015, World Bank. Standard errors in parentheses, adjusted for clustering in countries. Weighted by normalized student weights. * p<0.05, ** p<0.01, *** p<0.001.
